# Accidental Morcellation of Uterine Leiomyosarcoma Influences Relapse Free Survival but Does Not Negatively Influence Overall Survival

**DOI:** 10.3390/jcm12020591

**Published:** 2023-01-11

**Authors:** Verena M. C. Reichert, Zaher Alwafai, Marek T. Zygmunt, Marcus Vollmer, Günter Köhler

**Affiliations:** 1 Department of Obstetrics and Gynaecology, University Medicine Greifswald, Ferdinand-Sauerbruch-Straße, 17475 Greifswald, Germany; 2 Institute of Bioinformatics, University Medicine Greifswald, Felix-Hausdorff-Str. 8, 17475 Greifswald, Germany

**Keywords:** leiomyosarcoma, malignancy, morcellement, recurrence, survival

## Abstract

**Background:** Uterine leiomyosarcoma (LMS) is a rare entity amongst malignant gynaecological tumours and is mostly diagnosed after surgery for benign leiomyoma (LM) of the uterus. As minimal invasive surgery is widely used, the morcellation of LM and the uterus is rather common. As there is little known about the impact of the morcellation of LMS on local and distant metastases, as well as overall survival, we carried out a large-scale retrospective study. **Methods:** A total of 301 LMS cases from the German Clinical Competence Centre for Genital Sarcomas and Mixed Tumours were analysed. We distinguished morcellated and non-morcellated LMS from pT1 and >pT1 tumours. Fine–Gray competing risks regressions and cumulative incidence rates were computed for the time to local recurrence, distant metastases, and patient death. **Results:** The recurrence free interval in pT1 LMS was significantly lower in the morcellation group with a 2-year cumulative incidence rate of 49% vs. 26% in non-morcellated LMS (*p* = 0.001). No differences were seen in >pT1 tumours. Distant metastases were more frequently found in non-morcellated pT1 LMS compared to the morcellated cases (5-year cumulative incidence: 54% vs. 29%, *p* < 0.001). There was no significant difference in time to death between both groups neither in the pT1 stages nor in >pT1 disease. Subdistribution hazard ratios estimated by multivariable competing risks regressions for the morcellation of pT1 LMS were 2.11 for local recurrence (95% CI 1.41–3.16, *p* < 0.001) and 0.52 for distant metastases (95% CI 0.32–0.84, *p* = 0.008). **Conclusions:** Tumour morcellation is not associated with OS for pT1 tumours. The morcellation of pT1 LMS seems to prolong the time to distant metastases whereas local recurrence is more likely to occur after the morcellation of pT1 LMS.

## 1. Introduction

The majority of uterine masses occurring during the reproductive phase of women’s lives are leiomyoma (LM) also known as uterine fibroids [1]. The estimated incidence of LM is between 20% and 40% and is partially responsible for severe morbidity especially post menopause [2]. The symptoms associated with LM are menorrhagia, heavy menstrual bleeding with anaemia, dysmenorrhoea, lower abdominal pain, pelvic pressure, pelvic pain, and urinary incontinence, depending on the distinct location of the fibroid mass within the uterus [3]. The treatment options range from watchful waiting, hormonal and non-hormonal drugs such as NSAIDs, to more invasive options such as FUS (focused ultrasound therapy), uterine artery embolization, and radio frequency ablation, as well as minimally invasive techniques (e.g., hysteroscopic removal of the submucosal LM) with a myomectomy or a full hysterectomy at the other extreme. The management of symptomatic LM should be individually tailored to the patients’ symptoms, age, and the location of the fibroid tumour [3]. In postmenopausal women one of the leading symptoms of the existence of a fibroid tumour is known to be postmenopausal bleeding [3].

Those symptoms mentioned above could also indicate the presence of leiomyosarcoma (LMS) [4]. LMS are rare but highly aggressive malignancies of the uterus with an incidence rate of 0.36–0.86 in 100,000 women [5] accounting for 1–2% of all uterine malignancies [6]. Although most LMS are diagnosed at an early stage (60%) the 5-year survival rate throughout all stages of the disease varies from 25% to 76% [7]. When initially diagnosed in a metastatic state, the 5-year survival deteriorates to a poor 10–15% [7].

In order to estimate the risk of LMS prior to surgical treatment a preoperative leiomyosarcoma score (pLMS) was published by Köhler et al. [2]. This score allows for the assessment of the LMS risk and adequate surgical options for each patient [2]. However, prior to the existence of such a scoring system, there was much more uncertainty around diagnosis in this area, hence LMS was often surgically treated in the same way as LM [8]. The surgical treatment for LM could either be a total or supracervical hysterectomy or myomectomy. The use of power morcellators allow for a minimally invasive strategy in the treatment of myoma and have been applied for many years [9]. Unfortunately, because of the local dispersion of tissue throughout the entire abdominal cavity, the use of power morcellators may have fatal consequences when dealing with LMS [10].

Several studies and meta-analyses have reported a rate of 0.12‰ (1:8300) and 1.43‰ (1:700) of LMS treated as LM [11,12,13,14,15]. In 2015 Pritts et. al reported approximately 0.12 and 0.51 morcellated LMS per 1000 surgeries [13]. Hence it is a rare condition and associated with poor outcomes [16]. These results led to a conclusion that women had a higher risk of local recurrence and distant metastases in cases of morcellated malignant specimens [4], thus entering an incurable phase of the disease, as neither chemotherapy nor radiation therapy are effective treatment modalities. Additionally, inadequate surgical procedures are known to be associated with negative relapse-free and overall survival (OS) [16]. Due to small sample sizes, statistical significance is often missing and the cumulative incidence rates for local recurrence, distant metastases, and death at different tumour stages remain unclear.

In order to address this question, we carried out a retrospective study investigating the local and distant relapses in women with accidentally morcellated LMS or otherwise injured tumours during LMS surgery, both leading to a possible spread of malignant cells throughout the peritoneal cavity.

## 2. Material and Methods

### 2.1. Study Population

This register study included 301 LMS cases retrieved between 2005 and 2021 from the counselling database of the German Clinical Competence Centre for Genital Sarcomas and Mixed Tumours (DKSM, University Medicine Greifswald, Germany). Women underwent treatment for LM/LMS in various German in- and outpatient hospitals. All the patients included in this study gave written consent for data collection and for their use in a health study.

They were either treated primarily for LM and the final histological assessment revealed the presence of a LMS, or for LMS, in which case the latter was already pathologically proven by diagnostic procedures.

The mode of surgery was either minimally invasive or traditional abdominal. In 121 cases the tumour was injured or shredded, respectively, whereas 180 LM/LMS were kept intact during surgery. Morcellement and tumour injury took place predominantly during myomectomy or supracervical hysterectomy procedures.

The local recurrence was documented either as retroperitoneal pelvic and/or intraperitoneal pelvic/abdominal. Distant metastases also included parenchymal metastases in the liver, spleen, and stomach.

The analysis was performed separately for LMS stages pT1a/b (FIGO IA/B) (*n* = 229), defining a tumour limited to the uterus, and >pT1 (FIGO II-IVA), defining an additional extra-uterine pelvic and/or intraabdominal spread of the tumour (*n* = 72). Patients with primary distant metastases M1/FIGO IVB or with additional malignancies were excluded. Patients who received chemotherapy or radiotherapy were not excluded.

Several cases of local recurrences were detected by second-look laparoscopy or laparotomy with or without the removal of the residual cervix or uterus in cases of previous uterus-preserving surgery. The corresponding re-surgery took place between 2 and 6 months following the primary surgery. In the presented collective (pT1 and >pT1) 33.6% of morcellated and 9.3% of non-morcellated cases underwent a second surgery accordingly.

Follow-up was performed by the respective institutes according to the NCCN or German Guidelines. The data were regularly collected retrospectively every year by the DKSM recording the patients’ condition in terms of the relapse free interval (RFI) of local recurrence or distant metastases as well as OS. Death causes were checked and only a single competing cause of death was found.

Additional data were collected from the patients’ medical charts regarding age, tumour diameter and stage, ovariectomy, and menopausal status.

### 2.2. Statistical Analysis

A univariable statistical analysis of the continuous variables was carried out using the Student’s *t*-test and frequency distributions of the categorical variables were compared using Pearson’s Chi²-test without continuity correction. A time-to-event analysis was carried out using the Fine–Gray competing risks regression model. The event data includes the time to local recurrence in the pelvic, pelvic retroperitoneal, or intraabdominal location; time to distant metastases; and tumour-related mortality. Cumulative incidence rates were computed for both groups (morcellated vs. non-morcellated LMS) and included overall death as the only competing event for relapse events (local recurrence and distant metastases). Gray’s test was used to compare the cumulative incidence rates of the two LMS groups: with and without morcellation. Given the long time-period of surgeries, the follow-up time can be informative, and thus we limited the event time to 60 months after surgery for relapse events and 96 months for LMS death to reduce informative censoring. The cumulative incidence rates were computed with 95% confidence bands. A multivariable analysis was performed using competing risks regression for pT1 LMS cases only, starting with a full model that includes the following predictor variables: age, tumour diameter (as a factor variable giving 3 equally sized groups), menopause, morcellation, ovariectomy or adnexectomy. Using stepwise model selection with AIC, the models were further shrunk to the most meaningful feature sets. Fine–Gray subdistribution hazard ratios (SHR) with 95% confidence intervals were computed. A second regression was computed for pT1 LMS for each event type by adding the mitotic index, defined by the number of mitoses per 10 HPF, as a factor variable.

The proportional hazards assumption of each regression was checked by testing scaled Schoenfeld residuals. Multicollinearity was checked using variance inflation factors.

The statistical analysis was carried out using SPSS 27 (IBM Corp, Armonk, NY, USA) and R version 4.2.0 (statistical packages used: survival, tidycmprsk, MASS, The R Foundation for Statistical Computing, Vienna, Austria). The *p*-values are two-tailed and were considered statistically significant below 0.05.

## 3. Results

The patient and tumour characteristics are summarised in Table 1.

At stage pT1, patients with morcellation were statistically significantly younger and less likely to have had an ovariectomy (Table 1). There were no differences in the prognostically important variable of tumour diameter (*p* = 0.91) and in menopausal status (*p* = 0.20) between the two groups. Furthermore, the prognostically important mitotic index (mitoses/10 HPF) has been calculated, which also showed no significant differences (*p* = 0.63). At Stage >pT1 none of the previously mentioned variables showed any statistically significant differences.

Follow-up information was missing for 13 patients (7 at stage pT1, 6 at stage >pT1). The mean and median follow-up time for stage pT1 tumours in the non-morcellation group was 52.0 and 43.5 months (range 3–200), respectively, and 61.9 and 51.0 months (range 2–185), respectively, in the morcellation group. The mean and median follow-up time for stage >pT1 tumours in the non-morcellation group was 35.9 and 21.0 months (range 3–200), respectively, and 61.9 and 51.0 months (range 3–136), respectively, in the morcellation group. There were no statistically significant differences in both tumour stage groups (*p* = 0.23 and *p* = 0.60).

The number of mitoses per 10 HPF was not determined by the pathologist in 84 patients (56 at stage pT1, 28 at stage >pT1). The median number of mitoses per 10 HPF in pT1 LMS was 15, which defined the threshold for the mitotic index used in our risk regression comparison. The lower and upper quartiles were 10 and 25 for pT1 LMS. At stage >pT1 the quartiles were q_25_ = 10, q_50_ = 20, and q_75_ = 31.2. A total of 23 patients received adjuvant chemotherapy, 11 in the morcellated group (7 stage pT1, 4 stage >pT1) and 12 in the non-morcellated group (9 stage pT1, 3 stage >pT1). Due to the small number of postoperatively irradiated cases (12) in the entire cohort and its unproven efficacy, radiotherapy was not included in the event-time analysis.

**Figure 1:**
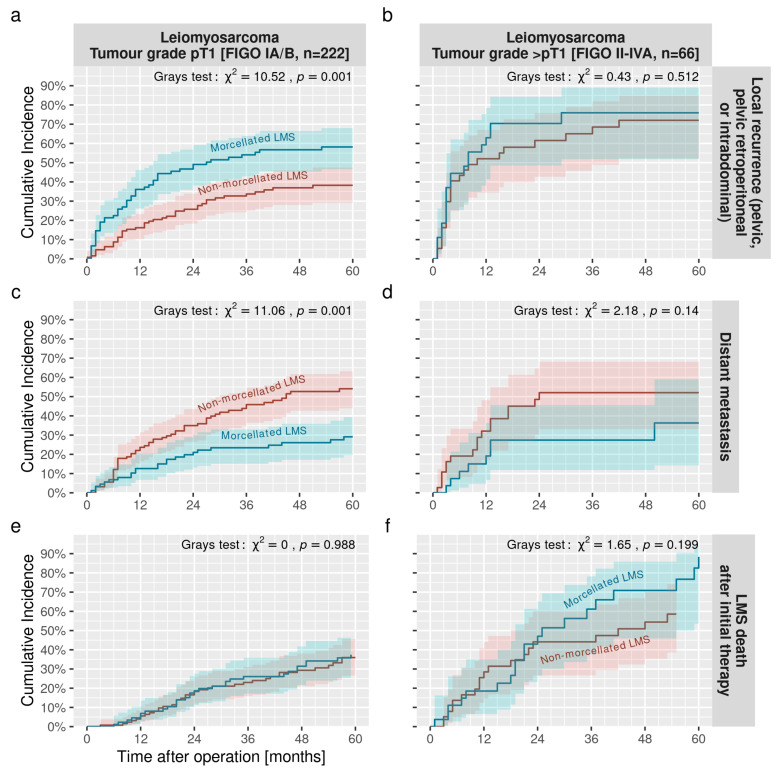
Cumulative incidence rates of the time to local recurrence (top), distant metastases (middle), and LMS mortality (bottom) are plotted in relation to LMS morcellation at stages pT1 (left panel (**a**,**c**,**e**)) and >pT1 (right panel (**b**,**d**,**f**)). Blue curves showing morcellated, red curves showing non-morcellated LMS with 95% confidence bands, respectively.

The 2-year cumulative incidence rate of local recurrence for stage pT1 LMS was 26% (95%-CI 18–34%) in non-morcellated LMS and was significantly higher in morcellated LMS (49%, 95%-CI 38–59%) (Figure 1a,b). In contrast, the 5-year cumulative incidence of distant metastases at stage pT1 was significantly higher in non-morcellated LMS compared to morcellated LMS (29% vs. 54%, Gray’s test: χ^2^ = 11.06, *p* < 0.001) (Figure 1c,d). Regardless of morcellement, the 5-year cumulative incidence rate of LMS death was 37% (95%-CI 29–44%) at stage pT1 and 71% (95%-CI 56–82%) at stage >pT1, (Figure 1e,f). Further incidence rates are summarised in Table 2 and the cumulative incidence rates for both tumour stages are plotted in Figure 1.

In the multivariable analysis, model reduction showed that local recurrence depends on the tumour diameter, menopause status, and morcellation (Table 3). Morcellation doubles the risk of local recurrence (SHR = 2.11, 95%-CI 1.41–3.16, *p* < 0.001) for stage pT1 LMS.

The cumulative incidence of distant metastases in stage pT1 LMS is demonstrated in Figure 1c (left panel). There were significant differences between both groups (Gray’s test *p* < 0.001) with a higher rate of distant metastases within the correctly treated group of non-morcellated LMS (Figure 1c). Patients with morcellated LMS survived longer without distant metastases, and respectively fewer distant metastases were reported in the shredded LMS group. In the multivariable analysis, distant metastases depended only upon morcellation and ovariectomy (Table 3). Morcellation halves the risk of distant metastases (SHR = 0.52, *p* = 0.008). Ovariectomies increases the risk (SHR = 1.76, *p* = 0.019). The same analyses for stage >pT1 LMS (Figure 1d) showed no significant differences between the two groups (*p* = 0.14).

Finally, we found no association between morcellation in stage pT1 LMS (Figure 1e) and LMS death (similar to overall death, as there were no other death causes documented before 96 months). There was no significantly different SHR whether LMS were accidentally morcellated or treated adequately during surgery. Hence, LMS death seems independent of the initial mode of surgical treatment. Only patient age showed a significantly increased risk (SHR = 1.33 for 10 year age difference, *p* = 0.006). However, when including the mitotic index, which reduces the number of patients studied (see sample numbers in Table 4), we do see changes in the coefficients and model composition. The influence of morcellation on the development of distant metastases is reduced and does not reach significance when adjusting for mitoses and ovariectomy (SHR = 0.64, *p* = 0.12). On the other hand, morcellation reached single-sided significance when analysing the time to LMS death when adjusting for mitoses and age (SHR = 1.69, *p* = 0.064).

## 4. Discussion

Since the widespread acceptance of minimally invasive surgery in the treatment of uterine LM and the concomitant application of power morcellators, there has been a certain number of accidentally morcellated LMS in the abdominal cavity. The effect on local recurrence rates, time to distant metastases and mortality remains questionable with only minimal data existing [17,18,19,20]. An online survey of the AAGL and ACOG members (2016) showed, that up to 75% of respondents had stopped the use of power morcellation during myomectomies and hysterectomies [21]. They believed that FDA warnings have not led to an improvement in patient outcomes but to the potential for harm to patients by choosing laparotomy over minimally invasive surgery in combination with the use of power morcellators [21]. In a safety communication the FDA adjusted its recommendations and supported the use of tissue containment systems when power morcellation is performed to isolate and contain the shredded tissue (https://www.fda.gov/medical-devices/surgery-devices/laparoscopic-power-morcellators, accessed on 17 June 2022).

To the best of our knowledge, this is the most comprehensive study to date investigating the consequences of the morcellation of uterine LMS in terms of local recurrence, distant metastases, and mortality, and furthermore taking the tumour stages into account, showing a separate analysis of stages, namely pT1 (*n* = 229) and >pT1 (*n* = 72).

In our analysis of stage pT1 LMS, patients without morcellation (*n* = 138) were statistically significantly younger and less likely to have had an ovariectomy than patients with morcellation (*n* = 91). Younger patients were more likely to have a myomectomy or supracervical hysterectomy with preservation of the ovaries. There were no differences for the prognostically important variable of tumour diameter (*p* = 0.67), menopausal status (*p* = 0.64), and mitotic index (*p* = 0.63). At stage >pT1, there were no differences for the variables of age, tumour diameter, ovariectomy, menopausal status, and mitotic index.

Regarding local relapses, Park et. al had included in their study 29 morcellated and 24 non-morcellated patients at stage pT1, and two morcellated and 1 non-morcellated stage pT2 cases. Due to the low number of cases, no separate analysis between the stages was carried out. Our data showed a significantly higher recurrence rate in the cases of morcellated LMS, which is in accordance with the data published by Park et al. in 2011 [19]. They investigated 56 patients mostly with stage pT1 LMS of the uterus; amongst these 31 (two >pT1) underwent total abdominal hysterectomy without morcellation and 25 (one >pT1) were treated differently, including tumour morcellation. No significant differences in the clinical and demographic variables were found between the two investigated groups. All the patients underwent a total abdominal hysterectomy after being diagnosed with LMS in a re-operation procedure but none of them were upstaged. Seven (22.6%) patients in the non-morcellated population and thirteen (52%) in the morcellation population had recurrent disease (*p* = 0.022) at the time of analysis [19]. Local recurrence within the peritoneal cavity appeared in 11 out of 13 patients. Park et al. referred to three former studies addressing the local recurrence of LMS after morcellation [10,20,22]. Morice et al. [20] showed that uterine morcellation was performed in 34 out of 123 cases of LMS without staging differentiation. The rate of local recurrence was higher after morcellation was performed than without morcellation, but the difference did not show any significance (3/34 = 8.8% vs. 3/82 = 3.7%; *p* = 0.25).

Another study by Einstein et. al [10] focused mainly on the completion surgery after the malignancy was proven by pathology in the specimen retrieved from the initially performed surgery [10]. Patients that underwent a supracervical hysterectomy without morcellation were not upstaged in contrast to 2/15 (13%) that were upstaged after completion surgery (in those cases morcellation was used) [10]. Another study, conducted by Perri et al. [22] investigated the treatment outcomes for LMS after morcellation. The study population was divided into two groups. Group A underwent a total abdominal hysterectomy without injury of the tumour or the macroscopic spread of malignant cells, and group B included only patients treated initially, for example by myomectomy or minimally invasive surgery such as a total laparoscopic hysterectomy using a power morcellator. The patients had similar demographic and clinical variables such as age, menopausal status, gravidity, and parity, as well as adjuvant treatments. The median follow-up time was 72 months for group A and 24 months for group B. The time to recurrence could not be related to the surgical technique used as the case numbers for each technique were too small for inferential statistics [22].

In our study the multivariable analysis shows that morcellation doubles the risk of local recurrence (SHR = 2.11, 95%-CI 1.41–3.16, *p* < 0.001) for stage pT1 LMS. Tumour status in addition to tumour diameter and postmenopausal status also had an influence (tumour diameter SHR = 1.26, 95%-CI 0.74–2.13, *p* = 0.4 comparing 8–11 cm with <8 cm tumour diameter; SHR = 1.98, 95%-CI 1.25–3.15, *p* = 0.004 comparing >11 cm with <8 cm tumour diameter; and for postmenopausal status SHR = 1.66, 95%-CI 1.08–2.53, *p* = 0.020). At stages >pT1, no effect on the local RFI was seen. This is understandable given the intrinsically greater likelihood of local spread in primary >pT1 tumours.

Regarding the incidence of distant metastases after surgery with and without tumour injury, only Park et al. [19] documented two patients with distant metastases in the morcellation group and three with distant metastases in the non-morcellation group. However, there was no separate analysis for the incidence of distant relapse. George et al. [23] performed a retrospective cohort study addressing recurrence-free survival (RFS) as well as OS in patients with uterine LMS. The study population was divided into patients that underwent a total abdominal hysterectomy (TAH) where the uterus was not injured and patients that underwent intraperitoneal morcellation of the tumour, including TAH, with injury to the tumour. The records of 58 patients were examined, 19 in the morcellation and 39 in the TAH group. Among these 34 recurrences were recorded, 14 in the morcellation cohort and 20 in the TAH cohort. Furthermore, the morcellation group had a significantly lower recurrence-free survival rate than the TAH group (10.8 vs. 39.6 months *p* = 0.002) [23]. No subgroup analysis (stage of disease) was made. These data suggest that intraperitoneal morcellation leads to a significant decrease in RFS. Abdominal and/or pelvic recurrence was more common in the morcellation group as compared to non-abdominal sites (*p* = 0.001). No survival data was available for distant metastases. A multivariate adjusted model demonstrated a more than three times increased risk of recurrence associated with morcellation (HR = 3.18; 95%-CI 1.5–6.8; *p* = 0.03) [23]. In the multivariable analysis of our data, distant metastases depend only on morcellation and ovariectomy (Table 3). Morcellation halves the risk of distant metastases (SHR = 0.52). At higher stages >pT1, no effect on distant RFI was evident.

Park et al. showed that six patients (19.4%) in their study died because of the disease in the non-morcellation group and eleven in the morcellation group (*p* = 0.046) leading to a 5-year OS rate of 60% in all patients, 73% in the non-morcellation group, and 46% in the morcellation group (*p* = 0.040). In a study conducted by Perri et al. [22] 18 out of 37 patients died of the disease, 8 without morcellation (8/21 = 38%) and 10 with morcellation (10/16 = 62.5%). Kaplan–Meier estimates showed significantly longer disease-free survival and better OS for the first group. The authors conclude that the only variable that significantly influenced survival was the mode of surgery. In addition to a very low number of cases, both studies contrast with our results, as we were able to show that OS is not affected, regardless of whether the LMS is injured during surgery and cells are spreading or not. However, taking mitoses into account, we have seen in a limited number of patients that morcellation has an increased, albeit non-significant, hazard ratio, which supports the results of the other authors. The influence of the mitotic index on OS has already been documented by Kyriazoglou et al. [24], whose study included 51 LMS cases and reached an adjusted hazard ratio of 3.28 while using the same threshold of 15 mitoses per 10 HPF as in our study. Different hazard ratios may be explained by the difference in FIGO stages: the proportion of FIGO II-IV in their study was 60.66% while we were computing the hazard ratios of FIGO I LMS only. When investigating OS, George et al. [23] showed a total number of 21 deaths (8 in the morcellation group, 13 in the TAH group). At 36 months, OS was 64% in the morcellation cohort and 73% in the TAH cohort. Poorer OS could also be assumed but did not reach a significant level due to the small numbers [23].

Our data from a comparatively large study cohort are consistent with the results on local/pelvic/peritoneal recurrence reported in the revised literature. There is a significantly higher rate of local recurrence when the cells of LMS were spread accidentally during surgery for pT1 LMS.

There is a lack of published data on the effect of morcellation on distant metastases. Only one publication was found [19] that mentioned distant metastases but without any statistical evidence. In summary, the data in the literature result from small samples and seem to be incomplete or contradictory. Furthermore, there is no information so far on the impact of morcellation on the time of the first occurrence of local and distant recurrences, as well as on the local and distant recurrence rate in higher stages of the disease.

Hence, we have demonstrated for the first time in a large patient cohort that morcellation of a postoperatively diagnosed uterine pT1 LMS has no negative impact on the occurrence of distant metastases or OS. However, the conclusions made from our study must be taken with caution as the cases from our LMS registry may not be representative. Furthermore, given the long study duration, we cannot rule out informed censoring (e.g., censoring depends on the operation date), and limitations exist due to the nature of the retrospective analysis, e.g., important factors may be missing or unsystematically recorded or documented.

## 5. Conclusions

In conclusion, in stages >pT1, no effect of morcellation on locoregional and distant RFI and OS is evident. It is remarkable that in stage pT1, morcellation doubles the locoregional recurrence risk and halves the risk of distant recurrence. The timing of the first occurrence of local recurrence does not change with morcellation, whereas morcellation surprisingly significantly delays the onset of distant metastases.

The “positive effect” of morcellation on distant relapses cannot be explained based on the variables studied. The potential effect of chemotherapy on this phenomenon could be excluded. The data may hypothetically suggest that morcellation with the spread of tumour cells in the peritoneal cavity may have a protective effect on the development of distant recurrences via yet unknown mechanisms.

OS is not altered by morcellation. This may be due to the differential, possibly mutually cancelling, effect of morcellation on local and distant recurrence. Overall, however, one must assume that the morcellation of uterine sarcomas must be considered an inadequate operation, even if OS is quite obviously not affected.

The number of mitoses or the mitotic index seems to be an important factor for all event types and should therefore be determined for each LMS. In particular, the impact of morcellation when adjusting for other prognostic factors for LMS death requires further research. Further prospective analysis must be conducted to confirm our findings.

## Figures and Tables

**Table 1:** Preoperative characteristics of patients without and with morcellation.

	Non-Morcellated LMS	Morcellated LMS	*p*-Value
**All** **stages (*n* = 301)**	*n* = 180 (59.8%)	*n* = 121 (40.2%)	
Age in years (mean)	54.3	51.9	0.06
Postmenopause	100 (55.6%)	64 (52.9%)	0.65
Ovariectomy	108 (60.0%)	61 (50.4%)	0.10
Tumour diameter cm (mean ± sd)	10.5 ± 6.0	10.7 ± 5.7	0.78
**pT1 (*n* = 229)**	*n* = 138 (60.3%)	*n* = 91 (39.7%)	
Age in years (mean)	53.6	50.1	0.015
Postmenopause	74 (53.6%)	41 (45.1%)	0.20
Ovariectomy	82 (59.4%)	37 (40.7%)	0.005
Tumour diameter cm (mean ± sd)	9.6 ± 5.1	9.5 ± 4.7	0.91
Number of mitoses per 10 HPF (mean ± sd)	20.7 ± 17.7	19.4 ± 18.6	0.63
**>pT1 (*n* = 72)**	*n* = 42 (58.3%)	*n* = 30 (41.7%)	
Age in years (mean)	56.8	57.4	0.79
Postmenopause	26 (61.9%)	23 (76.7%)	0.19
Ovariectomy	26 (61.9%)	24 (80.0%)	0.10
Tumour diameter cm (mean ± sd)	13.5 ± 7.8	14.2 ± 6.8	0.67
Number of mitoses per 10 HPF (mean ± sd)	19.1 ± 11.5	23.2 ± 16.9	0.54

**Table 2:** Cumulative incidence rates of leiomyosarcoma.

	Leiomyosarcoma Tumour Grade pT1				Leiomyosarcoma Tumour Grade >pT1			
Characteristic	*p*-Value ^1^	2-Year Cuminc	5-Year Cuminc	8-Year Cuminc	*p*-Value ^1^	2-Year Cuminc	5-Year Cuminc	8-Year Cuminc
**Local recurrence**								
Morcellation	0.001				0.5			
Non-morcellated LMS		26% (18%, 34%)	38% (29%, 47%)			62% (43%, 76%)	72% (52%, 85%)	
Morcellated LMS		49% (38%, 59%)	58% (47%, 68%)			70% (49%, 84%)	76% (52%, 89%)	
**Distant metastases**								
Morcellation	<0.001				0.14			
Non-morcellated LMS		35% (26%, 44%)	54% (44%, 63%)			52% (33%, 68%)	52% (33%, 68%)	
Morcellated LMS		21% (13%, 30%)	29% (20%, 39%)			27% (12%, 46%)	36% (14%, 59%)	
**LMS death**								
Overall		18% (13%, 24%)	37% (29%, 44%)	47% (39%, 55%)		45% (32%, 58%)	71% (56%, 82%)	81% (64%, 90%)
Morcellation	>0.9				0.2			
Non-morcellated LMS		18% (12%, 26%)	36% (27%, 45%)	48% (36%, 59%)		44% (27%, 60%)	59% (39%, 74%)	74% (50%, 88%)
Morcellated LMS		19% (11%, 28%)	37% (27%, 48%)	46% (33%, 57%)		47% (26%, 65%)	88% (56%, 97%)	88% 56%, 97%)

^1^ Gray’s Test.

**Table 3:** Results of the multivariable competing risks regression of pT1 LMS after stepwise model reduction showing the subdistribution hazard ratios (SHR) for each factor with 95%-confidence intervals for each event type, respectively.

	Local Recurrence (*n* = 216)			Distant Metastases (*n* = 216)			LMS Death (*n* = 222)		
Characteristic			SHR ^1^	95% CI ^1^	*p*-Value	SHR ^1^	95% CI ^1^	*p*-Value	SHR ^1^
Tumour diameter									
<8 cm			—	—					
8–11 cm			1.26	0.74, 2.13	0.4				
>11 cm			1.98	1.25, 3.15	0.004				
Menopause									
Premenopause	—	—							
Postmenopause			1.66	1.08, 2.53	0.020				
Morcellation									
Non-morcellated LMS	—	—		—	—				
Morcellated LMS	2.11	1.41, 3.16	<0.001	0.52	0.32, 0.84	0.008			
Ovariectomy									
No				—	—				
Yes				1.76	1.10, 2.82	0.019			
Age [10 years increment]							1.33	1.09, 1.63	0.006

^1^ SHR = Subdistribution Hazard Ratio, CI = Confidence Interval, *n* = number of complete samples.

**Table 4:** Results of the multivariable competing risks regression of pT1 LMS including mitoses counts after stepwise model reduction showing the subdistribution hazard ratios (SHR) of each factor with 95%-confidence intervals for each event type respectively.

	Local Recurrence (*n* = 164)			Distant Metastases (*n* = 164)			LMS Death (*n* = 167)		
Characteristic	SHR ^1^	95% CI ^1^	*p*-Value	SHR ^1^	95% CI ^1^	*p*-Value	SHR ^1^	95% CI ^1^	*p*-Value
Tumour diameter									
<8 cm	—	—							
8–11 cm	1.73	0.91, 3.30	0.094						
>11 cm	2.00	1.17, 3.40	0.011						
Menopause									
Premenopause	—	—							
Postmenopause	1.93	1.16, 3.21	0.012						
Morcellation									
Non-morcellated LMS	—	—		—	—				
Morcellated LMS	1.95	1.21, 3.14	0.006	0.64	0.37, 1.12	0.12	1.69	0.97, 2.95	0.065
Mitotic index									
Mitoses < 15 per 10 HPF	—	—		—	—		—	—	
Mitoses ≥ 15 per 10 HPF	1.43	0.89, 2.28	0.14	1.84	1.13, 3.00	0.015	1.61	0.93, 2.79	0.092
Ovariectomy									
No				—	—				
Yes				1.61	0.96, 2.71	0.071			
Age [10 year increment]							1.63	1.23, 2.15	<0.001

^1^ SHR = Subdistribution Hazard Ratio, CI = Confidence Interval, *n* = number of complete samples.

## Data Availability

Not applicable.
